# Mechanical forces suppress antiviral innate immune responses from asthmatic airway epithelial cells following rhinovirus infection

**DOI:** 10.1152/ajplung.00074.2022

**Published:** 2023-06-06

**Authors:** Punnam Chander Veerati, Andrew T. Reid, Kristy S. Nichol, Peter A. B. Wark, Darryl A. Knight, Nathan W. Bartlett, Christopher L. Grainge

**Affiliations:** ^1^School of Medicine and Public Health, University of Newcastle, Callaghan, New South Wales, Australia; ^2^Asthma and Breathing Research Program, Hunter Medical Research Institute, University of Newcastle, New Lambton Heights, New South Wales, Australia; ^3^Immune Health Program, Hunter Medical Research Institute, University of Newcastle, New Lambton Heights, New South Wales, Australia; ^4^Department of Respiratory and Sleep Medicine, John Hunter Hospital, New Lambton Heights, New South Wales, Australia; ^5^School of Biomedical Sciences and Pharmacy, University of Newcastle, Callaghan, New South Wales, Australia; ^6^Department of Anesthesiology, Pharmacology and Therapeutics, University of British Columbia, Vancouver, British Columbia, Canada; ^7^Research and Academic Affairs, Providence Health Care Research Institute, Vancouver, British Columbia, Canada

**Keywords:** antiviral innate immune responses, asthma, bronchoconstriction, mechanical forces, rhinovirus

## Abstract

Bronchoconstriction is the main physiological event in asthma, which leads to worsened clinical symptoms and generates mechanical stress within the airways. Virus infection is the primary cause of exacerbations in people with asthma, however, the impact that bronchoconstriction itself on host antiviral responses and viral replication is currently not well understood. Here we demonstrate how mechanical forces generated during bronchoconstriction may suppress antiviral responses at the airway epithelium without any difference in viral replication. Primary bronchial epithelial cells from donors with asthma were differentiated at the air-liquid interface. Differentiated cells were apically compressed (30 cmH_2_O) for 10 min every hour for 4 days to mimic bronchoconstriction. Two asthma disease models were developed with the application of compression, either before (“poor asthma control model,” *n* = 7) or following (“exacerbation model,” *n* = 4) rhinovirus (RV) infection. Samples were collected at 0, 24, 48, 72, and 96 h postinfection (hpi). Viral RNA, interferon (IFN)-β, IFN-λ, and host defense antiviral peptide gene expressions were measured along with IFN-β, IFN-λ, TGF-β_2_, interleukin-6 (IL-6), and IL-8 protein expression. Apical compression significantly suppressed RV-induced IFN-β protein from 48 hpi and IFN-λ from 72 hpi in the poor asthma control model. There was a nonsignificant reduction of both IFN-β and IFN-λ proteins from 48 hpi in the exacerbation model. Despite reductions in antiviral proteins, there was no significant change in viral replication in either model. Compressive stress mimicking bronchoconstriction inhibits antiviral innate immune responses from asthmatic airway epithelial cells when applied before RV infection.

**NEW & NOTEWORTHY** Bronchoconstriction is the main physiological event in asthma, which leads to worsened clinical symptoms and generates mechanical stress within the airways. Virus infection is the primary cause of exacerbations in people with asthma, however, the impact of bronchoconstriction on host antiviral responses and viral replication is unknown. We developed two disease models, in vitro, and found suppressed IFN response from cells following the application of compression and RV-A1 infection. This explains why people with asthma have deficient IFN response.

## Graphical abstract

**Figure:**
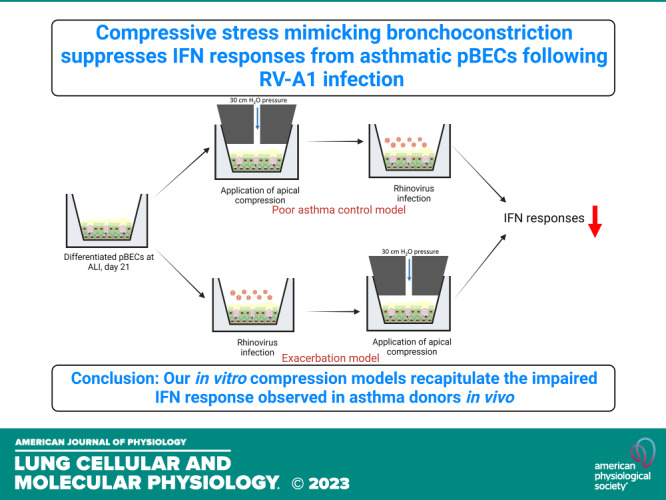

## INTRODUCTION

Asthma is a chronic respiratory disease with reversible airway narrowing (bronchoconstriction) caused by the contraction of airway smooth muscle. During bronchoconstriction, epithelial cells in the airway buckle and fold against each other generating compressive mechanical stress (1). This compressive stress appears to be sustained in the airway following a single constrictive stimulus in vivo (2). In several in vitro studies, application of a single continuous compressive stimulus was shown to activate epidermal growth factor receptors and release growth factors, cytokines, and mucus from airway epithelial cells (reviewed in Ref. 3). In addition, compression mimicking bronchoconstriction has been found to alter the mRNA signature of nonasthmatic airway epithelial cells toward that of the asthmatic epithelium (4).

Some people with asthma experience frequent exacerbations of symptoms including wheezing and shortness of breath, caused at least in part by bronchoconstriction (5). Viral infections, particularly rhinoviruses, are an important trigger of asthma exacerbations (6, 7). In nondiseased airways, rhinovirus (RV) infection induces antiviral responses from epithelial cells by stimulating interferon (IFN) production, thereby limiting viral replication, however, these responses appear to be deficient in the airways of some asthmatic populations (8–12). We have reported that innate immune responses are delayed in primary bronchial epithelial cells (pBECs) from patients with asthma following RV infection at low multiplicity of infection (MOI) (13). It remains unknown why airway epithelial cells from patients with asthma exhibit a reduction or delay in antiviral immunity; such a reduction would likely lead to prolonged viral infection and further worsening of their disease, as has been shown by the experimental RV infection of patients with asthma (14).

Patients with poorly controlled asthma, high symptom scores, and greater bronchial hyperresponsiveness are at greater risk of exacerbations; these individuals are also susceptible to viral infections (15–17). Patients with poorly controlled asthma, as well as patients with asthma who suffer viral airway infections, undergo bronchoconstriction simultaneously with a viral infection. Mechanical forces generated in the airway during repeated bronchoconstriction may influence epithelial antiviral responses following infection; how these two phenomena of infection and bronchoconstriction potentially influence each other has not been investigated. We hypothesize that compressive stress induced at the respiratory epithelium by bronchoconstriction suppresses innate antiviral responses leading to an increase in RV replication. Here, we aim to apply apical compression on fully differentiated asthmatic pBECs to mimic bronchoconstriction either before (a model of poorly controlled asthma) or after RV infection (viral infection-induced asthma exacerbation) and measure innate antiviral responses.

## MATERIALS AND METHODS

### Collection of Primary Bronchial Epithelial Cells and Ethics

All of the primary bronchial epithelial cells (pBECs) used in this study were from donors with asthma and further classified into mild to severe persistent disease, as defined by the Global Initiative for Asthma (GINA) (18). Participants had either never smoked or were former smokers; their clinical characteristics are shown in Table 1. The pBECs were obtained from bronchial brushings during bronchoscopy as previously described (12). Each participant provided written informed consent and all procedures were approved by the University of Newcastle (H-163-1205) and Hunter New England Area Health Service (05/08/10/3.09) ethics committees.

**Table 1.** Clinical characteristics of asthma donors used in both compression models

	Poor Asthma Control Model	Exacerbation Model
Number, *n*	7	4
Severity (*n*)	Mild (5)	Severe (4)
	Moderate (1)	
	Severe (1)	
Age, yr (SD)	59.9 (14)	65.8 (9.8)
Male, *n* (%)	5 (71.4)	2 (50)
Smoking history (Former/Never)	3/4	2/2
FEV1, % predicted (SD)	82.4 (8.6)	45 (13.9)
FVC, % predicted (SD)	89.4 (9.1)	66.8 (10.4)
(FEV1/FVC) % (SD)	70.5 (6.8)	57.5 (15.7)
BAL cell count (E/N/M/P)	3/3/0/1	1/1/1/1
Atopy (SPT positive)	3	1
ICS dose, BDP† equivalent, mg (SD)	325 (114.6)#	425 (129.9)##

† ICS dose was adjusted to beclomethasone (BDP) equivalent. #Six out of seven donors were on ICS in the poor asthma control model. ##All four donors were on ICS in the exacerbation model. BAL, bronchoalveolar lavage; E, eosinophilic; FEV1, forced expiratory volume in 1 s; FVC, forced vital capacity; ICS, inhaled corticosteroid; M, mixed; N, neutrophilic; P, paucigranular; SPT, skin prick test.

### Air-Liquid Interface Culture

Following pBEC collection, cells were maintained in a bronchial epithelial cell growth medium (BEGM; Lonza, Switzerland). To differentiate pBECs at air-liquid interface (ALI) culture, cells were seeded on 0.4 µm pore transwell membranes (Corning) and grown for 21 days for full differentiation with media changes on alternate days as previously described (19). Transepithelial electrical resistance (TEER) was measured every 7th day, post ALI establishment (*day 0*), to evaluate barrier integrity.

### Application of Apical Compression on pBECs to Mimic Bronchoconstriction

Apical compression of well-differentiated pBECs was performed as previously described (20, 21). In addition to the earlier established setup, a custom-designed programmable logic controller (PLC) unit was equipped with electrically controlled valves (The Lee Company, Essex, CT) and operated with preloaded commands to allow user-defined periods of compressive (30 cmH_2_O) and atmospheric pressure delivered to the cells (see Supplemental Fig. S1; https://doi.org/10.6084/m9.figshare.19217625).

### Infection of ALI Cultures with RV

Human rhinovirus minor group (RV-A1) was used for these experiments. RV-A1 stocks were diluted to MOI 0.001 with starvation media [BEBM containing 1% Insulin-Transferrin-Selenium (ITS) and 0.5% Linoleic Acid (LA)] and added to ALI cultures apically for 6 h as previously described (13). Cells were harvested at 24-h intervals following RV-A1 infection.

### Modeling “Poor Asthma Control” and “Exacerbation” with Apical Compression and RV Infection

**Figure 1.**
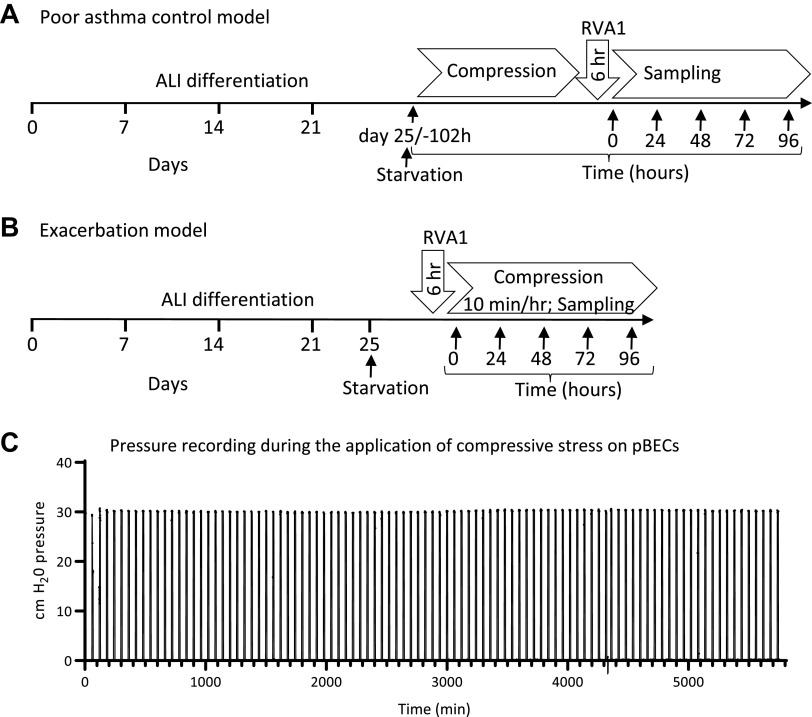
Modeling apical compression, in vitro. *A*: poor asthma control model: Asthmatic air-liquid interface (ALI) cultures exposed to repeated apical mechanical stress mimicking poor asthma control, followed by rhinovirus (RV) infection. *B*: exacerbation model: asthmatic ALI cultures infected with RV followed by repeated apical mechanical stress mimicking virally induced asthma exacerbation. *C*: representative manometer (Gastools, Australia) recording of 30 cmH_2_O pressure on differentiated pBECs during the application of repeated apical compression. pBECs, primary bronchial epithelial cells.

We developed two compression models, the first to mimic poor pre-existent asthma control followed by viral infection (poor asthma control model), and the second mimicked bronchoconstriction following a virally induced asthma exacerbation (exacerbation model; Fig. 1, *A* and *B*). In the poor asthma control model, well-differentiated ALI cultures were exposed to apical compression by air at 30 cmH_2_O pressure for 10 min every hour for 96 h (Fig. 1*C*; representative manometer recording) to mimic repeated bronchoconstriction, followed by RV-A1 infection with MOI 0.001 [infection procedure as previously described (13)]. In the exacerbation model, a similar RV-A1 infection was performed, followed by an identical compression regimen. We chose this compression regimen to mimic repeated bronchoconstriction experienced by an individual with asthma with short-acting bronchodilator treatment.

### Extracellular Protein Measurement

Apical and basal media, sampled from ALI cultures, were analyzed for released proteins using ELISA. IFN-β (PBL Assay Science), IFN-λ1/3, IL-6, and IL-8 (R&D systems) were measured from apical media, and total TGF-β_2_ (R&D systems) release was measured from basal media as per manufacturer’s instructions.

### RNA Extraction, RT-PCR, and qPCR

Total RNA was extracted from lysed cells using miRNeasy mini kit (Qiagen, Netherlands). RNA was reverse transcribed using a high-capacity cDNA reverse transcription kit (Applied Biosystems). Absolute quantification of gene expression was performed on ABI 7500 Fast Real-time PCR system (Applied Biosystems) using customized primers and probes for viral RNA and IFNs, LL-37, and secretory leukocyte protease inhibitor (SLPI) genes and normalized to 18S rRNA as previously described (22; Supplemental Table S1; https://doi.org/10.6084/m9.figshare.19217622).

### Statistical Analysis

Data were analyzed using GraphPad Prism 9.00 software (La Jolla, CA). Paired data between two groups at a time point were analyzed using a two-tailed Wilcoxon matched-pairs signed-ranks test. Paired data for more than two groups at a time point were analyzed using the Friedman test with Dunn’s multiple comparisons. *P* values < 0.05 were considered statistically significant.

## RESULTS

### Poor Asthma Control Model: Apical Compression Suppresses IFN Protein Production from Asthmatic pBECs following RV-A1 Infection, with No Difference in RV-A1 Replication

**Figure 2.**
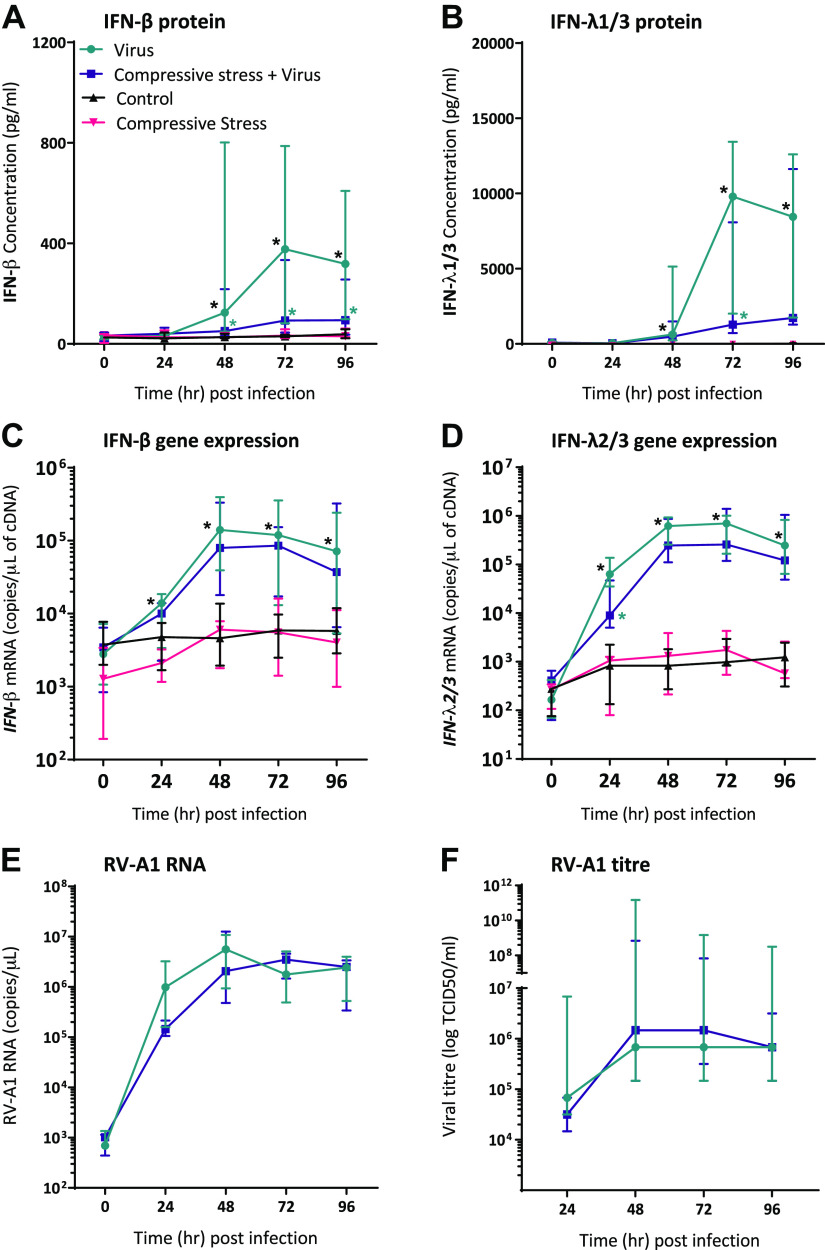
Application of apical compression before viral infection suppresses IFN proteins and genes from differentiated asthmatic pBECs with no change in viral replication. Time course of virus-induced IFN-β (*A*) and IFN-λ1/3 (*B*) proteins measured from apical supernatants, IFN-β (*C*) and IFN-λ2/3 (*D*) gene expression measured from complementary DNA (cDNA) from asthmatic pBECs. Time course of viral RNA (*E*) and viral titer (*F*) was measured from cDNA and apical supernatants from asthmatic pBECs. Data represented as median (IQR) with sample size, *n* = 7. Paired data analysis was performed among two groups at a given time point using a two-tailed Wilcoxon matched-pairs signed-rank test. Color-matched asterisks were used to represent the significance of the groups compared. For example, black color asterisks represent virus-induced IFN responses in comparison with noninfected control at a given time point, and green asterisks are for compression effect on virus-induced IFN response in comparison with virus alone group. *P < 0.05 is considered significant. IFN, interferon; pBECs, primary bronchial epithelial cells.

Fully differentiated asthmatic pBECs (TEER measurements are shown in Supplemental Fig. S2, *A* and *B*; https://doi.org/10.6084/m9.figshare.21579633) were infected with RV-A1, MOI 0.001. There was a significant induction of IFN-β and IFN-λ proteins from fully differentiated asthmatic pBECs following RV-A1 infection when compared with the noninfected group at 48, 72, and 96 hpi (Fig. 2*A*, *B*). Peak IFN-β and IFN-λ protein was detected at 72 hpi in the virus alone group. Application of compression before virus infection significantly suppressed IFN-β protein levels at 48, 72, and 96 hpi (*P* = 0.0313, *P* = 0.0469, *P* = 0.0469, respectively) and IFN-λ at 72 hpi (*P* = 0.0313; Fig. 2, *A* and *B*; individual virus-infected IFN protein data points at 48, 72, and 96 hpi are shown in Supplemental Fig. S3, *A* and *B*; https://doi.org/10.6084/m9.figshare.19217628). A similar trend was observed with IFN gene expression, RV-A1 infection significantly induced *IFN-β* and *IFN-λ* gene expression from 24 hpi compared with the noninfected group and with peak mRNA copy numbers at 48 and 72 hpi, respectively (Fig. 2, *C* and *D*). Application of prior apical compression significantly reduced *IFN-λ* gene expression at 24 hpi (*P* < 0.0313; Fig. 2*D*; individual gene expression data points from 24 to 96 hpi shown in Supplemental Fig. S3*D*; https://doi.org/10.6084/m9.figshare.19217628). Though there was a decrease in *IFN-β* gene expression with the application of compression, there was no significant difference among the virus-infected groups at any time point (Fig. 2*C*; individual gene expression data points from 24 to 96 hpi shown in Supplemental Fig. S3*C*; https://doi.org/10.6084/m9.figshare.19217628). Despite the difference in IFN responses with apical compression, there was no significant difference in viral RNA copy number or viral titer between either virus-infected group (with and without prior compression; Fig. 2, *E* and *F*). The peak viral RNA in the virus-alone group was observed at 48 hpi and in the prior compression group at 72 hpi before it stabilized at 96 hpi (Fig. 2*E*). As there was no difference in viral RNA copy numbers among the virus-infected groups, we measured the expression of host defense antiviral peptides such as cathelicidin peptide (LL-37) and secretory leukocyte protease inhibitor (SLPI). There was no induction of LL-37 and SLPI gene expression following RV-A1 infection compared with noninfected controls at any time point (Supplemental Fig. S4, *A* and *B*; https://doi.org/10.6084/m9.figshare.22350988).

### Poor Asthma Control Model: Apical Compression Did Not Alter Inflammatory Responses or Basal TGF-β_2_ Release from Asthmatic pBECs

**Figure 3.**
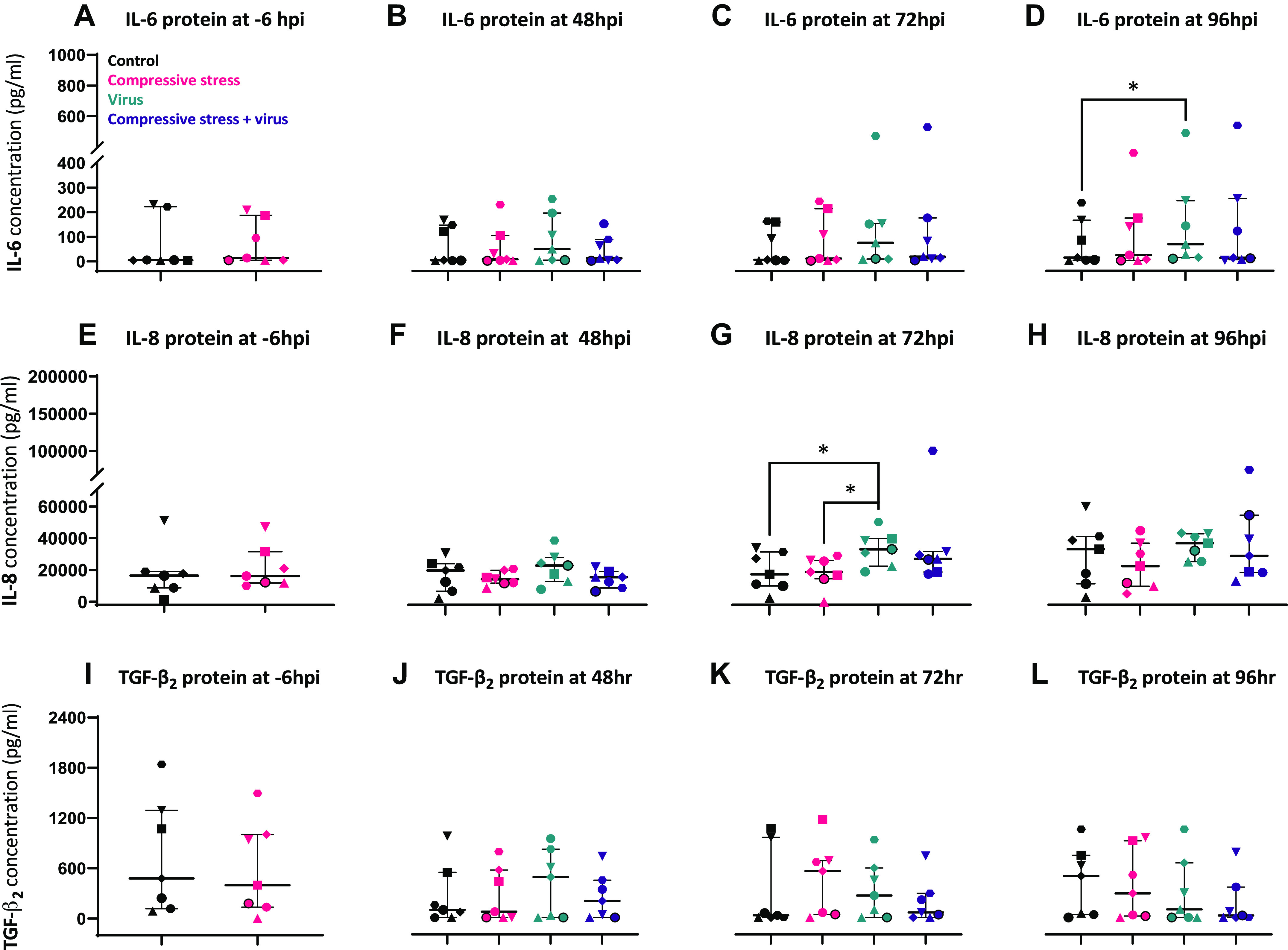
Application of apical compression before viral infection has no effect on the release of inflammatory mediators or TGF-β_2_ protein from pBECs. IL-6 (*A*), IL-8 (*E*), and TGF-β_2_ (*I*) protein release from pBECs following the application of apical compression and IL-6 (*B–D*), IL-8 (*F–H*), and TGF-β_2_ (*J–L*) following RV-A1 infection. Sample size, *n* = 7 and data represented as median (IQR). Paired data analyzed using two-tailed Wilcoxon matched-pairs signed-rank test (*A*, *E*, *I*) and paired multiple groups data using Friedman test with Dunn’s multiple comparisons at each time point, *P < 0.05 was considered significant. A separate symbol is assigned for each donor to compare among the groups. pBECs, primary bronchial epithelial cells.

Following 96 h of apical compression, inflammatory mediators [interleukin-6 (IL-6) and IL-8] were measured from the pBEC apical supernatant. There was no difference in the release of IL-6 or IL-8 following compression alone compared with controls (Fig. 3, *A* and *E*). Following RV-A1 infection, there was a significant induction of IL-6 protein in the virus-alone group at 96 hpi compared with respective control (Fig. 3, *B*–*D*; *P* < 0.029). IL-8 protein concentrations increased from 0 to 96 hpi in all groups. However, apical IL-8 concentration further increased following RV-A1 infection from 48 to 96 hpi with a significant increase at 72 hpi compared with control (*P* = 0.0389) and compression alone (*P* = 0.0113) groups (Fig. 3, *F*–*H*). There was no difference in the basolateral release of TGF-β_2_ protein from asthmatic differentiated pBECs following 4 days of apical compression (Fig. 3*I*) or following RV-A1 infection (Fig. 3, *J*–*L*).

### Exacerbation Model: Apical Compression Induces a Trend toward Suppression of IFN Protein Production from pBECs but Did Not Affect RV-A1 Replication

**Figure 4.**
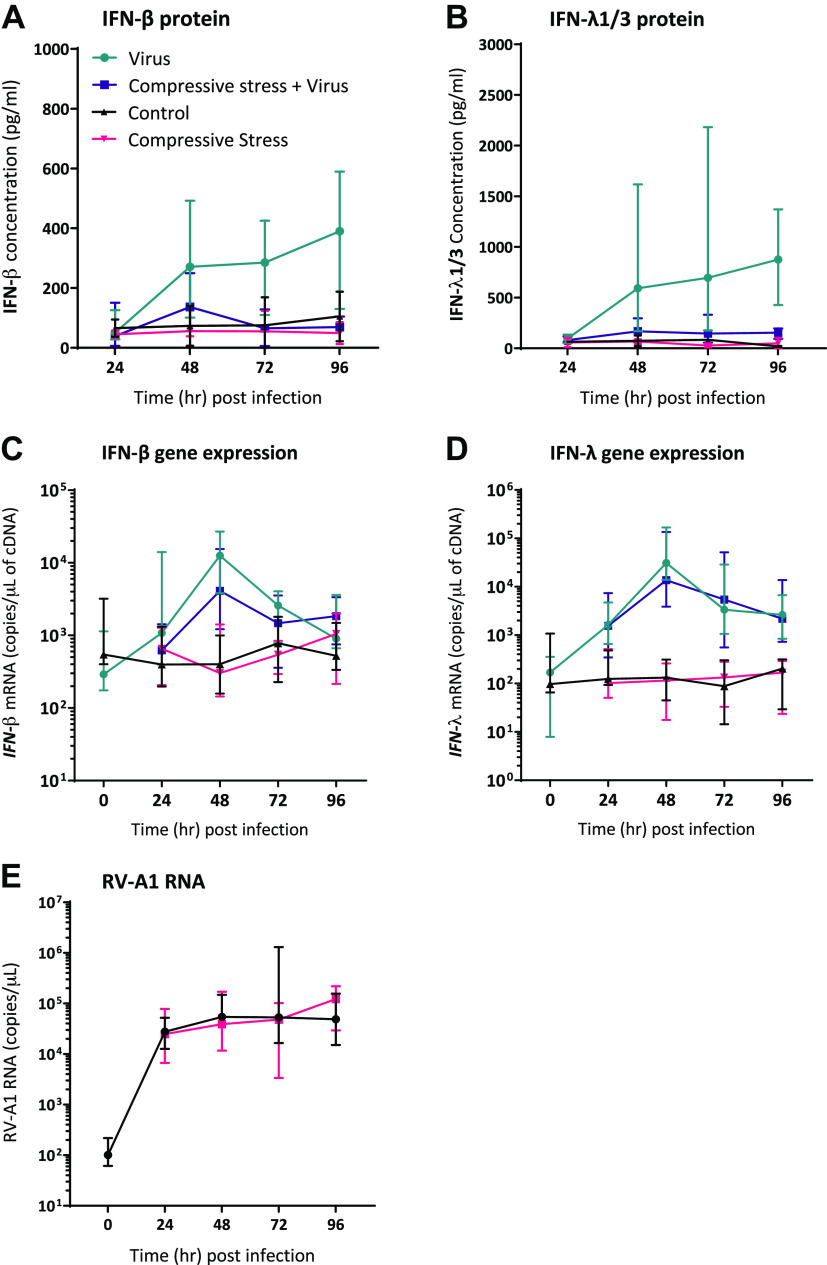
Application of apical compression following RV-A1 infection appears to suppress IFN responses from differentiated asthmatic pBECs with no effect on viral replication. Time course of virus-induced IFN-β (*A*) and IFN-λ1/3 (*B*) proteins measured from apical PBS washes during harvest from asthmatic pBECs. IFN-β (*C*), IFN-λ (*D*), and viral RNA copy numbers (*E*) were measured from cDNA from asthmatic pBECs. Sample size, *n* = 4 and data represented as median (IQR). Only virus infected groups, virus alone, and compression + virus groups were analyzed using a two-tailed Wilcoxon matched-pairs signed-rank test at each time point, *P* < 0.05 was considered significant. pBECs, primary bronchial epithelial cells.

Similar to the poor asthma control model, both IFN-β and IFN-λ proteins were induced from fully differentiated asthmatic pBECs following RV-A1 infection (Fig. 4, *A* and *B*); TEER in Supplemental Fig. S2, *C* and *D* (https://doi.org/10.6084/m9.figshare.21579633). Peak IFN-β and IFN-λ proteins were detected at 96 hpi in the virus-alone group. Application of compression following RV-A1 infection suppressed both IFN-β and IFN-λ protein concentrations at 48, 72, and 96 hpi, though these reductions did not reach statistical significance (Fig. 4, *A* and *B*; individual virus-infected IFN protein data points at 48, 72, and 96 hpi are shown in Supplemental Fig. S5, *A* and *B*; https://doi.org/10.6084/m9.figshare.19217616). RV-A1 infection-induced *IFN-β* and *IFN-λ* gene expression from 24 hpi with peak mRNA copy numbers at 48 hpi (Fig. 4, *C* and *D*). Application of apical compression following viral infection did not alter *IFN-β* or *IFN-λ* gene expression (Fig. 4, *C* and *D*; individual gene expression data points from 24 to 96 hpi shown in Supplemental Fig. S5, *C* and *D*; https://doi.org/10.6084/m9.figshare.19217616). Similar to the poor asthma control model, application of apical compression did not alter the viral RNA copy numbers following infection (Fig. 4*E*), there was also no induction of LL-37 and SLPI expression following RV-A1 infection (Supplemental Fig. S4, *C* and *D*; https://doi.org/10.6084/m9.figshare.22350988).

### Exacerbation Model: Apical Compression Did Not Alter Inflammatory Responses or Basal TGF-β_2_ Release from Asthmatic pBECs

**Figure 5.**
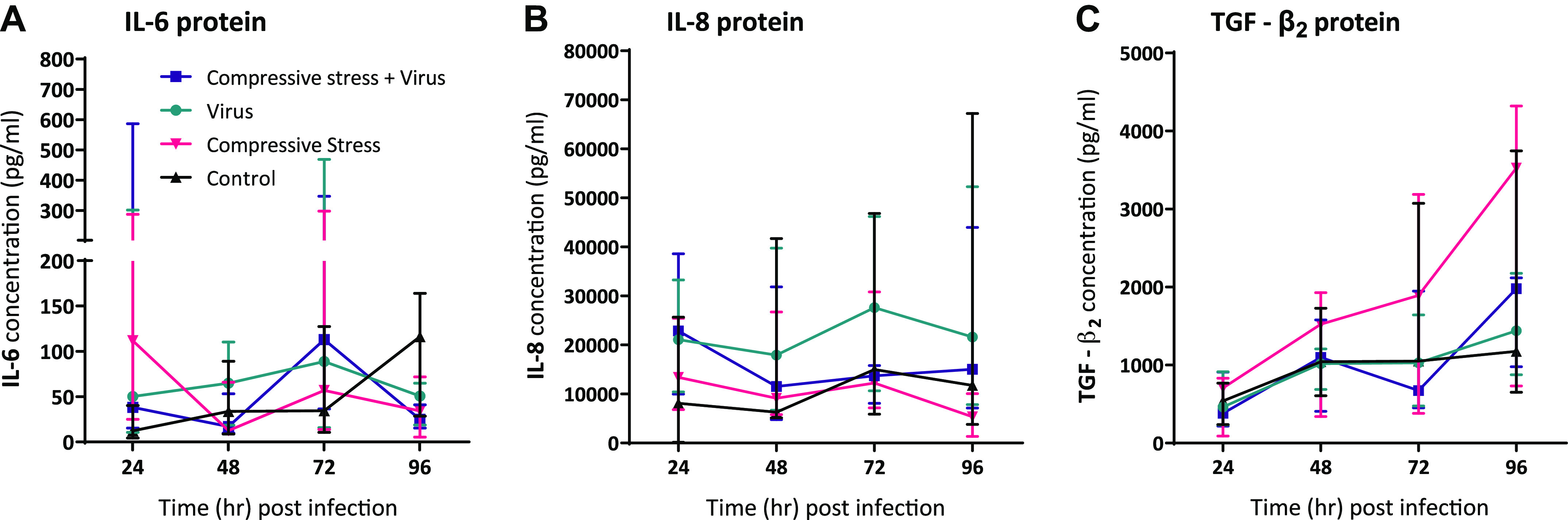
Application of apical compression following viral infection has no effect on the release of inflammatory mediators or TGF-β_2_ protein from pBECs. Time course of apical IL-6 (*A*), apical IL-8 (*B*), and basal TGF-β_2_ (*C*) protein release from pBECs following RV-A1 infection and apical compression. Sample size, *n* = 4, data represented as median (IQR). Paired multiple groups data were analyzed using Friedman test with Dunn’s multiple comparisons at each time point, *P* < 0.05 was considered significant. pBECs, primary bronchial epithelial cells.

Following RV-A1 infection and apical compression, both inflammatory mediators (IL-6 and IL-8) at each time point were measured from apical PBS washes. There was no difference in the release of IL-6 or IL-8 with compression and viral infection (Fig. 5, *A* and *B*). The basolateral release of TGF-β_2_ protein was not different between groups (Fig. 5*C*).

## DISCUSSION

In asthma, ∼50% of virally induced asthma exacerbations are caused by RV infections (23) and during such exacerbations, airways constrict and generate compressive stress within the epithelium (1). The epithelial response to viral infection in conjunction with compressive stress has not been investigated previously. Here we report that antiviral innate immunity is suppressed by the application of compressive stress mimicking mechanical stress induced in vivo by bronchoconstriction. We modeled two scenarios pertinent to asthma using differentiated pBECs from asthma donors coupled with physiologically relevant RV-A1 infection (13). We modeled poorly controlled asthma using repeated apical compression events on cells followed by infection with RV-A1. We also modeled virus-induced bronchoconstriction by infecting cells with RV-A1 and then applying repeated apical compression. In the poor asthma control model, we found a reduction in IFN-β and IFN-λ protein along with reduced *IFN-β* and *IFN-λ* gene expression. There was a trend toward a reduction in IFN-β and IFN-λ protein in the exacerbation model.

To mimic repeated bronchoconstriction in vitro, we applied apical compression of 30 cmH_2_O pressure [estimated mechanical stress during maximal bronchospasm (1)] on ALI differentiated pBECs, 10 min every hour for 4 days. This is in contrast to previous studies using a single continuous apical compression for 1–8 h (4, 20, 24–33). We chose this approach to better mimic poorly controlled asthma or viral-induced asthma exacerbations with repeated bronchoconstriction with treatment by short-acting bronchodilators.

A previous study modeling continuous compression of 8 h reported a significant induction of TGF-β_2_ protein from healthy pBECs after 16 h of compression (25). We found no induction of TGF-β_2_ protein either immediately after our intermittent compression regimen (as in poor control model) or over the whole 96 h timecourse following RV infection (in both models). The lack of TGF-β_2_ induction may reflect the difference in compression regimen or the fully differentiated cells that we used in our models. The previous continuous compression studies were performed in differentiating (*day 14*) rather than fully differentiated cells (*day 21*); in addition, cells were mostly obtained from nonasthma donors (25, 29, 30, 32). Current data suggest that 21 days of ALI are essential for pBECs to achieve full differentiation with formed tight intercellular junctions, mucus secretion, and cilia movement (34, 35).

Regarding viral replication in our models, there was similar viral replication kinetics in the virus-alone group in the poor control model compared with our previous reports using the same virus and MOI (13, 35). In the exacerbation model, viral replication in RV alone group was twofold lower compared with the poor asthma control model and lower than our previous reports (13, 35). This could be due to the patient’s disease severity (severe patient with asthma) and the difference in the experimental plan. To compress the cells in the exacerbation model, they could not be incubated with apical media; PBS washes were performed only at times of harvest. Thus, there was no potential for the accumulation of virus/proteins within apical cell surface fluid. However, with the application of apical compression either before (as in poor asthma control model) or after (as in exacerbation model) RV-A1 infection, did not influence viral replication kinetics.

Following RV-A1 infection, we measured IFN-β and IFN-λ gene and protein expression from asthmatic pBECs. In both models, application of compressive stress suppressed IFN proteins from pBECs following infection. In the poor asthma control model, both IFN-β and IFN-λ proteins were significantly suppressed along with *IFN-λ* gene expression. In the exacerbation model, active compression led to a reduction in both IFN protein and gene expression but this did not reach statistical significance. Previously, we have reported that innate immune responses from pBECs from asthmatic donors are delayed when compared with healthy cells using RV-A1 (MOI 0.001) infection with the same postinfection sampling time points (13). Multiple previous studies have also reported deficient IFN responses from asthma pBECs following RV infection when compared with healthy cells (8–10, 12). However, many of these studies used high MOI in undifferentiated monolayer basal pBECs which do not mimic the airway epithelium. Deficient or lower IFN responses may potentially increase the risk of exacerbations with persistent lower respiratory tract infections. In addition, it has been reported that a lower IFN response favors coinfections and increases the hospitalization of people with asthma (36). In addition to IFN responses, we measured the host defense antiviral peptides LL-37 and SLPI, which limit the virus replication and control the spread of nonenveloped viruses (37–39). However, in our study models, there was no induction of antiviral peptide expression following RV infection at any given time point indicating no influence on viral replication.

Our experimental models may explain why people with asthma have deficient or delayed IFN responses following viral infections, although an investigation of the mechanisms linking compression with reduced innate immunity was outside the scope of this work. Our study was limited by sample size, cell numbers, and the use of a single RV strain, each of which can be addressed by additional experimental work.

In summary, we were successful in modeling bronchoconstriction and RV infection together in well-differentiated pBECs grown at ALI mimicking asthma exacerbations and poorly controlled asthma. Our findings suggest that compressive stress significantly suppresses IFN responses from asthmatic pBECs but does not impact RV replication following low MOI RV-A1 infection in the poor control model and show a trend toward suppression in exacerbation model; these data may explain why people with asthma appear to have impaired antiviral responses. Studies focused on regaining this reduction of IFN response from pBECs may identify new therapeutic targets to treat individuals with asthma and its exacerbations.

## DATA AVAILABILITY

Data will be made available upon reasonable request.

## SUPPLEMENTAL DATA

10.6084/m9.figshare.19217625 Supplemental Fig. S1: https://doi.org/10.6084/m9.figshare.19217625.

10.6084/m9.figshare.21579633 Supplemental Fig. S2: https://doi.org/10.6084/m9.figshare.21579633.

10.6084/m9.figshare.19217628 Supplemental Fig. S3: https://doi.org/10.6084/m9.figshare.19217628.

10.6084/m9.figshare.22350988 Supplemental Fig. S4: https://doi.org/10.6084/m9.figshare.22350988.

10.6084/m9.figshare.19217616 Supplemental Fig. S5: https://doi.org/10.6084/m9.figshare.19217616.

10.6084/m9.figshare.19217622 Supplemental Table S1: https://doi.org/10.6084/m9.figshare.19217622.

## GRANTS

This work was funded by the Australian National Health and Medical Research Council (NHMRC—https://www.nhmrc.gov.au/; G1700343) and the Royal Australasian College of Physicians.

## DISCLOSURES

Nathan Bartlett is an editor of *American Journal of Physiology-Lung Cellular and Molecular Physiology* and was not involved and did not have access to information regarding the peer-review process or final disposition of this article. An alternate editor oversaw the peer-review and decision-making process for this article. None of the other authors has any conflicts of interest, financial or otherwise, to disclose.

## AUTHOR CONTRIBUTIONS

P.C.V., D.A.K., N.W.B., and C.L.G. conceived and designed research; P.C.V. and K.S.N. performed experiments; P.C.V. and C.L.G. analyzed data; P.C.V., A.T.R., P.A.B.W., D.A.K., N.W.B., and C.L.G. interpreted results of experiments; P.C.V. prepared figures; P.C.V. and A.T.R. drafted manuscript; P.C.V., A.T.R., K.S.N., P.A.B.W., D.A.K., N.W.B., and C.L.G. edited and revised manuscript; P.C.V., A.T.R., K.S.N., P.A.B.W., D.A.K., N.W.B., and C.L.G. approved final version of manuscript.
